# The Inherited Blindness Protein AIPL1 Regulates the Ubiquitin-Like FAT10 Pathway

**DOI:** 10.1371/journal.pone.0030866

**Published:** 2012-02-07

**Authors:** John S. Bett, Naheed Kanuga, Emma Richet, Gunter Schmidtke, Marcus Groettrup, Michael E. Cheetham, Jacqueline van der Spuy

**Affiliations:** 1 UCL Institute of Ophthalmology, London, United Kingdom; 2 Division of Immunology, Department of Biology, University of Constance, Konstanz, Germany; Instituto de Investigación Hospital 12 de Octubre, Spain

## Abstract

Mutations in AIPL1 cause the inherited blindness Leber congenital amaurosis (LCA). AIPL1 has previously been shown to interact with NUB1, which facilitates the proteasomal degradation of proteins modified with the ubiquitin-like protein FAT10. Here we report that AIPL1 binds non-covalently to free FAT10 and FAT10ylated proteins and can form a ternary complex with FAT10 and NUB1. In addition, AIPL1 antagonised the NUB1-mediated degradation of the model FAT10 conjugate, FAT10-DHFR, and pathogenic mutations of AIPL1 were defective in inhibiting this degradation. While all AIPL1 mutants tested still bound FAT10-DHFR, there was a close correlation between the ability of the mutants to interact with NUB1 and their ability to prevent NUB1-mediated degradation. Interestingly, AIPL1 also co-immunoprecipitated the E1 activating enzyme for FAT10, UBA6, suggesting AIPL1 may have a role in directly regulating the FAT10 conjugation machinery. These studies are the first to implicate FAT10 in retinal cell biology and LCA pathogenesis, and reveal a new role of AIPL1 in regulating the FAT10 pathway.

## Introduction

Mutations in the retina and pineal-specific aryl hydrocarbon receptor interacting protein-like 1 (AIPL1) lead to the inherited blindness Leber congenital amaurosis (LCA), which is characterised by severe vision loss or blindness at birth [1]. AIPL1 has been proposed to act as a specialized chaperone for the cGMP phosphodiesterase PDE6 [2], [3], [4] and interacts with Hsp70 and Hsp90 family members to form a chaperone heterocomplex [5], but the precise role of AIPL1 in the retina has yet to be fully elucidated. AIPL1 was also reported to interact with NEDD8 ultimate buster-1 (NUB1) [6], which promotes the proteasomal degradation of the ubiquitin-like modifiers (UBLs) NEDD8 and FAT10 and their modification targets [7], [8], thus implicating AIPL1 in photoreceptor protein degradation pathways. AIPL1 has previously been shown to modulate NUB1 nuclear translocation and suppress the aggregation of NUB1 fragments [9], but the precise functional relationship between these two proteins has remained unknown.

Modification of proteins by ubiquitin and UBLs controls a diverse array of cellular processes through altering protein interactions, function and degradation [10]. Conjugation of UBLs to their targets is a multi-step process involving several sequential steps. Firstly, an E1 activating enzyme adenylates the conserved C-terminal diglycine motif of the UBL, followed quickly by the formation of a high-energy thioester between the UBL and the E1 active-site cysteine. The charged UBL is then passed to the active-site cysteine of a specific E2 conjugating enzyme to form a second thioester bond. Finally, the E2 enzyme coordinates with a substrate-bound E3 ligase to covalently conjugate the UBL onto an internal lysine in the substrate through an isopeptide bond [10].

FAT10 is a member of the family of UBL modifiers [11]. It contains two UBL domains separated by a short linker, with 29% and 36% identity to ubiquitin respectively, and is conjugated onto a lysine residue in the target protein through its C-terminal diglycine motif [11]. FAT10 is a ubiquitin-independent signal for proteasomal degradation [12], [13], but other than autoFAT10ylation of the recently characterised FAT10 E2 enzyme USE1 [14], the physiological substrates for FAT10 modification are currently unidentified. NUB1 and NUB1L (a longer splice variant of NUB1) were found to interact with and promote the proteasomal degradation of FAT10 and FAT10-modified proteins [7], [15]. NUB1L contains three tandem ubiquitin-associated (UBA) domains toward its C-terminus and a single UBL domain near the N-terminus, whereas NUB1 lacks 14 amino acids in the second UBA domain such that it has only two UBA domains. NUB1 and NUB1L have been shown to bind the 26S proteasome through their single UBL domain and bind FAT10 through their UBA domains, and thus act on the proteasome to facilitate the degradation of FAT10-modified proteins [15]. Indeed, NUB1L has been shown to be essential for the degradation of FAT10-fusion proteins *in vitro* [13].

While the E1 activating enzyme for FAT10 has been identified as UBA6 (E1-L2) [16] and USE1 has recently been described as an E2 conjugating enzyme [14], no other components of the FAT10 conjugation machinery or physiological substrates have thus far been reported, other than autoFAT10ylation of USE1 [14]. A role for FAT10 in the mammalian immune system has been proposed as it is enriched in the thymus and spleen [17], its expression is induced by IFN-gamma and TNF-alpha, and FAT10 knockout mice display hypersensitivity to endotoxin challenge [18]. FAT10 has also been implicated in apoptosis [11], various cancers [19], [20] as well as in the cellular response to misfolded protein accumulation [21], but the precise physiological functions of FAT10 have not yet been demonstrated. FAT10 has also been shown to interact with, and prevent, MAD2 association with kinetochores [17], [22], suggesting the biological role of FAT10 modification may extend beyond protein degradation.

Here, we report that the photoreceptor-specific protein AIPL1 is a novel regulator of the FAT10 pathway. We demonstrate that AIPL1 acts to antagonise NUB1-mediated degradation of the model FAT10 substrate FAT10-DHFR, and that pathogenic mutations are defective in this function. In summary, these studies highlight a previously unidentified role of the FAT10 pathway in photoreceptor cell biology with implications in the pathogenesis of LCA.

## Materials and Methods

### Antibodies

Rabbit polyclonal antibodies to AIPL1, NUB1 and FAT10 were described previously [9], [12], [23], [24], [25]. Mouse monoclonal anti-HA (purified immunoglobulin, H3663), anti-polyhistidine (ascites fluid, H1029), anti-FLAG (purified immunoglobulin, F3165) and anti-c-Myc (purified immunoglobulin, M4439) antibodies were purchased from Sigma (St. Louis, MO), and goat polyclonal anti-GST antibody was purchased from GE Healthcare (Amersham, UK). Horseradish peroxidise-conjugated mouse, rabbit and goat secondary antibodies were purchased from Pierce Biotechnology (Rockford, IL).

### Plasmids

Expression plasmids p-GEXT-2T-AIPL1 (GST-AIPL1), pCMV-Tag3C-AIPL1 (Myc-AIPL1) and pCMV-Tag3C-AIPL1 mutants A197P, C239R and G262S were described previously [9], [23], as were HA-FAT10-pCDNA3.1, HA-FAT10-AV-DHFR [11], [12], His_6_-UBA6-pCDNA3.1 and His_6_-3XFLAG-FAT10ΔGG [14]. NUB1-p3XFLAG-CMV (NUB1-FLAG) was generated by subcloning NUB1 from pEGFP-C1-NUB1 [9] into p3XFLAG-CMV-14 vector (Sigma, St. Louis, MO) using standard methods.

### Cell culture and transfection

SK-N-SH neuroblastoma cells (purchased from ATCC) were maintained and grown as previously described [26]. Cells were transfected 24 hours after seeding in six-well plates, using Lipofectamine and Plus reagent according to manufacturer's instructions (Invitrogen, Paisley, UK). Single wells were transfected as indicated with 900 ng HA-FAT10, His-FLAG-FAT10ΔGG or HA-FAT10-DHFR, and 300 ng of NUB1-FLAG, Myc-AIPL1/mutants and His-UBA6, and empty plasmid DNA was used to maintain a total plasmid DNA transfection amount of 1.5 µg where necessary. Treatment of cells with 50 µM proteasome inhibitor MG132 (Enzo Life Sciences, Plymouth, UK) was performed 20 hours post-transfection for 4 hours. Cycloheximide (Sigma, St Louis, MO) treatment was performed at 50 µg/ml final concentration 20–24 hours post-transfection for 0–4 hours.

### Immunocytochemistry

SK-N-SH cells were transfected as described above. Cells were washed with PBS 24 hours post-transfection, fixed with 4% paraformaldehyde for 15 min, and permeabilised in 0.1% Triton-X-100 for 5 min. Cells were incubated in block solution (10% donkey serum in PBS) for 30 min, followed by incubation with primary antibody anti-HA (1∶150) (Sigma, St Louis, MO) in block solution for 1 h. Primary antibody was removed followed by 4×5 min washes in PBS, prior to incubation with Cy2-conjugated anti-mouse secondary (1∶150) in block solution for 45 min. Secondary antibody was removed and cells were washed 4 times in PBS. Fluorescence was visualised using a Zeiss LSM 510 laser scanning confocal microscope.

### Western blotting and immunoprecipitation

Twenty-four hours post-transfection, cells were washed in PBS and lysed in DM buffer (1% n-dodecyl-β-D-maltoside; 10 mM EDTA; protease inhibitor cocktail (Sigma, St Louis, MO) in PBS) (300 µl/well) for 15 min at 4°C. Cell lysates were denatured with 3× sample buffer (62.5 mM Tris-HCl, pH 6.8; 2.5% SDS; 5% 2-mercaptoethanol; 10% glycerol; 0.25% bromophenol blue) and 15 µl resolved by denaturing SDS-PAGE. Transfer onto nitrocellulose membrane and western blotting was carried out as previously described [24]. For immunoprecipitation, 250 µl of cell lysate was incubated with 2 µl of antibody for 2 hours at 4°C, followed by addition of 50 µL of 50% slurry Protein G Sepharose 4 Fast Flow (GE Healthcare, Amersham, UK) equilibrated in DM buffer. Following overnight incubation at 4°C, the immune complex was thoroughly washed in ice-cold DM buffer (5×1 ml washes), and proteins eluted in 50 µl sample buffer and analysed by western blot analysis. Western blotting and immunoprecipitation results were reproduced from between 2 and 7 independent replicates per experiment. Numerical values documenting observed fold changes were calculated using standard densitometric analyses (ImageJ) from at least 3 independent experimental replicates. Specifically, the intensity of the bands was measured from 3 independent experiments (ImageJ) and the resultant data normalised to the experiment-specific internal control (1 or 100%) to calculate the fold change or change in percentage respectively. For the cycloheximide experiment, the Wilcoxon signed-rank test was used to calculate the level of significance of the temporal change in FAT10 levels from 3 independent experiments (UCLA Statistics Online Computational Resource).

### Recombinant protein purification and pull-down experiments

Expression and purification of GST and GST-AIPL1 from *Escherichia coli* JM109 cells was performed as previously described [5]. For pull-down of HA-FAT10 from transfected SK-N-SH lysates, cells were harvested from individual wells of a six-well plate as described above and 250 µl lysate was pre-cleared with 10 µg GST for 2 h at 4°C. GST was removed from lysates by the addition of 50 µl of 50% slurry Glutathione-Sepharose 4B (GE Healthcare, Amersham, UK). Pre-cleared lysates were then incubated with either 0.38 nmol GST or 0.38 nmol GST-AIPL1 at 4°C overnight. Bound proteins were pulled down using 50 µl of 50% slurry Glutathione-Sepharose 4B, and washed thoroughly in ice-cold PBS (5×1 ml washes) before elution in 50 µl sample buffer and western blot analysis. For pull-down of recombinant purified His_6_-FAT10 (Enzo Life Sciences, Plymouth, UK), 0.38 nmol of GST or GST-AIPL1 was incubated with 0.38 nmol His_6_-FAT10 in 50 mM Tris-HCL pH 6.8 at 4°C overnight, and bound proteins were pulled down as described. Pull-down experiments were reproduced in five independent experiments.

## Results

### AIPL1 alters the NUB1-mediated degradation of FAT10

**Figure 1:**
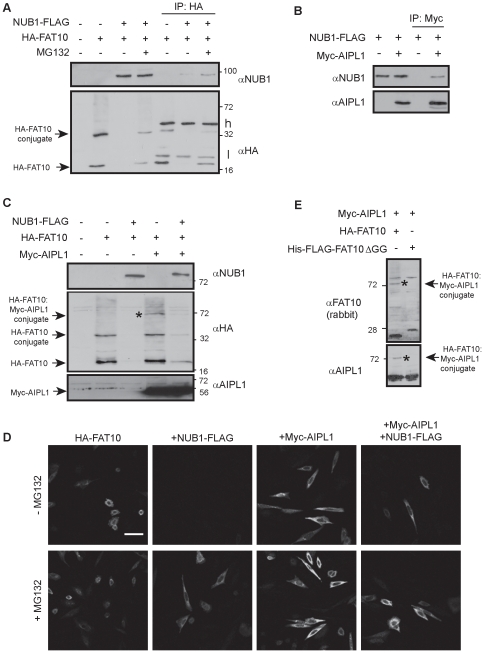
AIPL1 alters the NUB1-mediated degradation of FAT10. SK-N-SH Cells were transfected with NUB1-FLAG, HA-FAT10 and Myc-AIPL1 vectors in the presence and absence of the proteasome inhibitor MG132, as indicated. Cell lysates were harvested 24 hours post-transfection and immunoprecipitates were analyzed by immunoblotting to detect the protein indicated. (A) NUB1 interacts with FAT10 and accelerates the degradation of free FAT10 and FAT10-modified proteins. The change in levels of FAT10 was measured from 3 independent experiments (n = 3) of duplicate samples. Heavy (h) and light (l) immunoglobulin chains are indicated. (B) NUB1 co-precipitates with AIPL1. (C) AIPL1 enhances the steady-state levels of free FAT10 and FAT10 modified proteins, both alone and in the presence of NUB1. The change in levels of FAT10 was measured from 5 independent experiments (n = 5) of duplicate samples. (D) HA-FAT10 was visualised by immunocytochemical analysis with anti-HA and Cy2-conjugated secondary antibody. NUB1-mediated degradation of FAT10 is altered by the presence of AIPL1. Scale bar is 20 µM. (C) and (E) A small proportion of AIPL1 is itself covalently modified with FAT10, as detected by anti-HA, anti-AIPL1 and anti-FAT10 (rabbit polyclonal) antibodies. The percentage of AIPL1 modified by FAT10 was measured from 3 independent experiments (n = 3). Conjugation of FAT10 to AIPL1 is prevented using a FAT10 diglycine deletion mutant. The position of molecular weight markers is indicated in kilodalton (kDa).

NUB1, and its longer splice variant NUB1L, have previously been shown to interact with and elicit the proteasomal degradation of free FAT10 and FAT10-modified proteins [7], [15]. To confirm these findings, SK-N-SH cells [9] were transfected with HA-FAT10 alone or with NUB1-FLAG in the presence and absence of the proteasome inhibitor MG132. Western blot analysis revealed a FAT10 expression profile of unconjugated FAT10, an unidentified FAT10-conjugated protein migrating at ∼36 kDa and a characteristic smear of high molecular weight FAT10-modified proteins (Figure 1A). Co-expression of NUB1 with FAT10 resulted in the disappearance of all FAT10 species, while proteasome inhibition reduced the NUB1-mediated degradation of FAT10 to ∼70% (73%±9) of FAT10 levels alone, in agreement with NUB1's role in the proteasomal degradation of FAT10. To confirm the well characterised interaction between FAT10 and NUB1, co-immunoprecipitation experiments were performed on cell lysates expressing FAT10 and NUB1. Immunoprecipitation of HA-FAT10 with anti-HA antibody confirmed that NUB1 interacts with FAT10, and this interaction was enhanced ∼5-fold when the proteasome was inhibited with MG132 (Figure 1A). NUB1 has been previously shown to interact with the inherited blindness protein AIPL1 [6], and we confirmed that NUB1-FLAG co-precipitated with myc-AIPL1 (Figure 1B) before assessing what effect AIPL1 has on the NUB1-mediated degradation of FAT10. SK-N-SH cells were transfected with FAT10 alone and in combination with either AIPL1 or NUB1, then with both AIPL1 and NUB1. Interestingly, western blot analysis revealed that co-expression of AIPL1 with FAT10 caused an ∼30% (30%±9) increase in monomeric FAT10 and a similar increase in high molecular weight FAT10-conjugated proteins, suggesting that AIPL1 interacts with the FAT10 degradation pathway (Figure 1C). AIPL1 also affected the NUB1-mediated degradation profile of FAT10, whereby the steady-state levels of monomeric FAT10 and the ∼36 kDa FAT10 conjugate were increased by ∼6 fold and ∼5-fold respectively in the presence of AIPL1 and NUB1, compared to NUB1 alone (Figure 1C). This suggested AIPL1 may block the NUB1-mediated degradation of FAT10ylated proteins. Similarly, immunocytochemical analysis also revealed the NUB1-mediated proteasomal-targeting of HA-FAT10, and the inhibition of this NUB1-mediated FAT10 degradation by AIPL1 (Figure 1D). In denaturing western blots, an additional prominent HA-FAT10 band of around 72 kDa was also evident in the presence of AIPL1 and was detectable by AIPL1, HA and FAT10 antibodies, suggesting that a minor proportion of AIPL1 itself (3.5%±1) may be modified by FAT10 (Figure 1C and E, asterisk). To confirm that this was a covalent conjugate of AIPL1-FAT10 mediated through the C-terminal diglycine motif of FAT10, we expressed the FAT10 mutant His-FLAG-FAT10ΔGG and found that AIPL1 could not be modified with this version of FAT10 under denaturing conditions (Figure 1E). Therefore, not only does AIPL1 appear to block NUB1-mediated degradation of FAT10ylated proteins, but a small proportion of AIPL1 (<5%) itself is modified with FAT10.

### AIPL1 directly binds to FAT10 and interacts with high molecular weight FAT10ylated proteins

**Figure 2:**
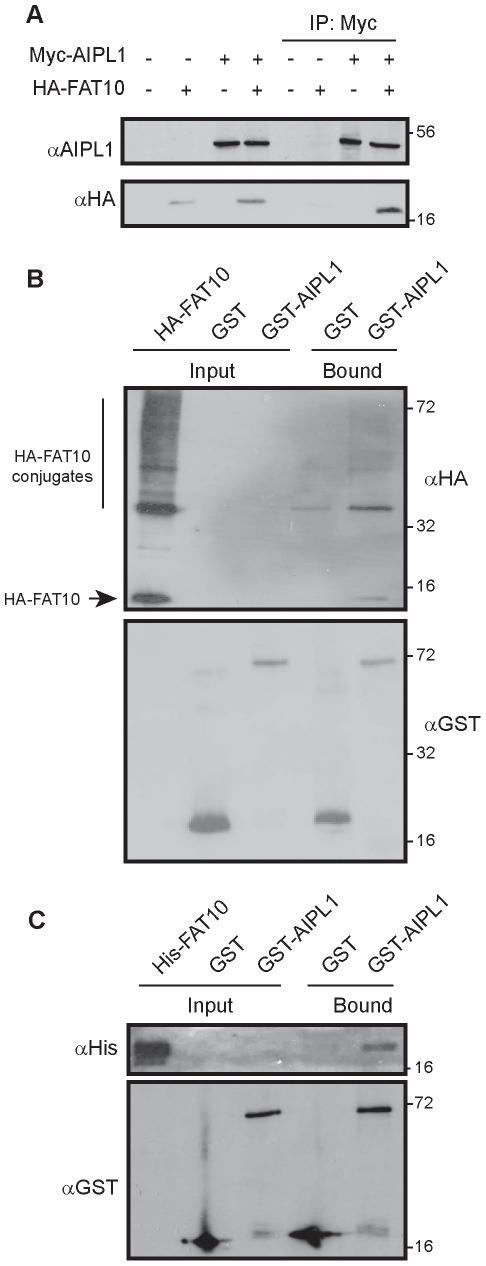
AIPL1 binds to FAT10 and FAT10-modified proteins. (A) Monomeric FAT10 co-precipitates with AIPL1 from transfected SK-N-SH cell lysates. Cells were transfected with the indicated constructs and immunoprecipitation was performed followed by immunoblot analysis. (B) Recombinant purified GST-AIPL1 but not GST can pull down free FAT10 and FAT10-conjugated proteins from HA-FAT10-transfected cell lysates. (C) Recombinant purified GST-AIPL1, but not GST, pulls down recombinant purified His6-FAT10. The position of molecular weight markers is indicated in kilodalton (kDa).

The finding that AIPL1 increases steady-state levels of FAT10 and FAT10-modified proteins led us to investigate whether AIPL1 also interacts non-covalently with FAT10 itself. To address this, AIPL1 was co-expressed with FAT10 and co-precipitation experiments were performed. Monomeric FAT10 co-precipitated specifically with AIPL1 (Figure 2A), supporting the hypothesis that AIPL1 is in a complex with FAT10. The ability of AIPL1 to interact non-covalently with FAT10 was not dependent on the C-terminal diglycine motif of FAT10, as AIPL1 could also co-precipitate a conjugation-deficient His-FLAG-FAT10ΔGG mutant (data not shown). As AIPL1 was capable of interacting with unconjugated FAT10, it was of interest to test whether AIPL1 would also associate with high molecular weight FAT10-modified proteins. To test this hypothesis, glutathione-S-transferase (GST) pull-down experiments were performed on the lysates of SK-N-SH cells transfected with HA-FAT10 plasmid. GST-AIPL1, but not GST, was able to pull-down free FAT10 as well as a range of high molecular weight FAT10 species, confirming the ability of AIPL1 to interact with FAT10-modified proteins (Figure 2B). To investigate if AIPL1 could bind directly to FAT10, GST pull-down experiments were performed using equimolar amounts of recombinant purified GST-AIPL1 and His_6_-FAT10. His_6_-FAT10 was specifically pulled down by GST-AIPL1, confirming that AIPL1 and FAT10 do interact directly (Figure 2C).

### AIPL1, NUB1 and FAT10 form a ternary complex

**Figure 3:**
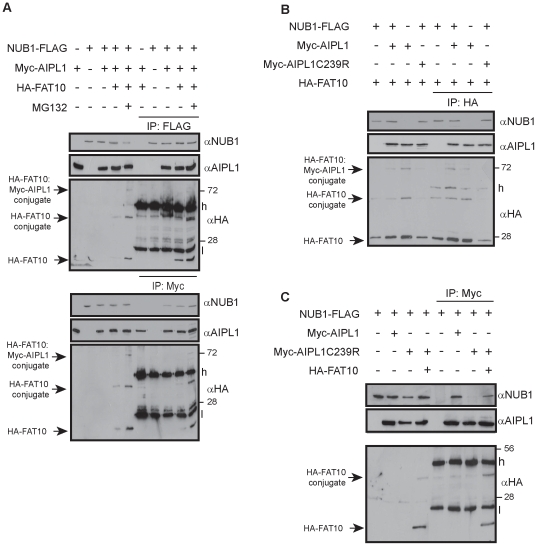
AIPL1, FAT10 and NUB1 form a ternary complex. Cells were transfected with constructs as indicated followed by immunoprecipitation and immunoblot analysis. (A) AIPL1 and FAT10 both co-precipitate with NUB1 (top panel); NUB1 and FAT10 both co-precipitate with AIPL1 (bottom panel). MG132 increased FAT10 steady-state levels and the amount of co-precipitated AIPL1 (top panel) or NUB1 (bottom panel). (B) NUB1 and AIPL1 both co-precipitate with FAT10. The AIPL1 C239R pathogenic mutant immunoprecipitated FAT10, but did not affect its NUB1-mediated degradation profile. (C) The AIPL1 C239R mutant did not immunoprecipitate NUB1 but FAT10 expression promoted their interaction. Heavy (h) and light (l) immunoglobulin chains are indicated. The position of molecular weight markers is indicated in kilodalton (kDa).

As AIPL1 binds to both NUB1 [27] (Figure 1) and FAT10 (Figure 2), we sought to investigate whether AIPL1, NUB1 and FAT10 could form a ternary complex. To test this, combinations of AIPL1, NUB1 and FAT10 were expressed in SK-N-SH cells followed by co-precipitation and western blotting. AIPL1 co-precipitated with NUB1 (Figure 3A, top panel) and vice versa (Figure 3A, bottom panel), and this reciprocal interaction was unaffected by FAT10 which precipitated with both AIPL1 and NUB1 (Figure 3A). Treatment of cells with proteasome inhibitor prevented degradation of FAT10 in the presence of NUB1 and increased the amount of FAT10 that co-precipitated with NUB1 and AIPL1 by ∼2 fold and ∼5 fold respectively. Moreover, proteasome inhibition increased the amount of AIPL1 that co-precipitated with NUB1 by ∼40%, and vice versa in the reciprocal interaction precipitation (Figure 3A). Immunoprecipitation of FAT10 co-precipitated both AIPL1 and NUB1 (Figure 3B), showing that regardless of which protein (AIPL1, NUB1 or FAT10) was immunoprecipitated, the other two proteins always co-precipitated. The mutation of cysteine 239 to arginine (C239R) in AIPL1 causes LCA and prevents AIPL1 from binding to NUB1 [27]. This mutant was still able to bind FAT10 (Figure 3B) and therefore could be used to test if FAT10 restores the association of AIPL1 C239R to NUB1 through ternary complex formation. Myc-tagged AIPL1 C329R was immunoprecipitated and shown to be defective in its interaction with NUB1 (Figure 3C) in agreement with previous studies [27]. However, expression of FAT10 stimulated NUB1 co-precipitation with AIPL1 C239R, strongly suggesting the three proteins form a ternary complex (Figure 3C). Interestingly, the C239R mutant was unable to alter the NUB1-mediated degradation profile of FAT10 (Figure 3B), suggesting the direct interaction of AIPL1 with NUB1 is important in modifying FAT10 degradation.

### AIPL1 antagonises the NUB1-mediated degradation of FAT10-DHFR

**Figure 4:**
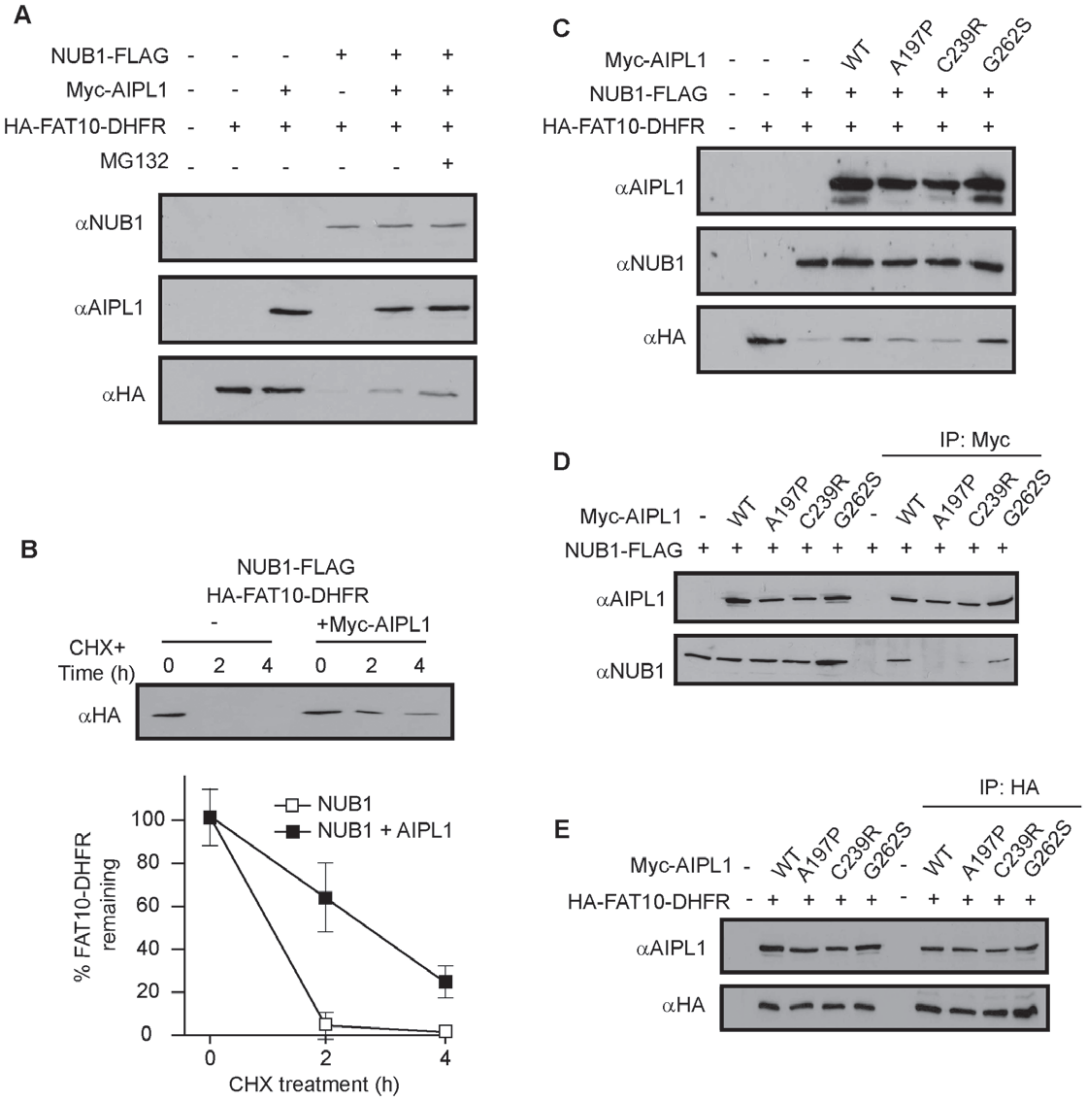
AIPL1 interacts with NUB1 to block the degradation of FAT10-DHFR. Cells were transfected with the indicated constructs, then treated 24 hours later with cycloheximide (CHX) to block protein synthesis and assess degradation of FAT10-DHFR over the indicated times. (A) AIPL1 blocked the NUB1-mediated degradation of FAT10-DHFR, and the effect was stronger in the presence of MG132. (B) AIPL1 delayed the degradation of FAT10-DHFR in the presence of NUB1. The percentage of FAT10 remaining was measured from 3 independent experiments (n = 3), and the level of significance calculated using the Wilcoxon signed-rank test. (C) Pathogenic AIPL1 mutants A197P and C239R were defective in blocking FAT10-DHFR degradation, while the G262S mutant was able to block degradation. (D) NUB1 co-precipitated with both WT and G262S AIPL1, but not with the A197P or C239R mutants. (E) FAT10-DHFR co-precipitated with WT AIPL1, and the mutants A197P, C239R and G262S.

AIPL1 increased the steady-state levels of FAT10 and FAT10-modified proteins, which suggested that AIPL1 may protect against the NUB1-mediated degradation of FAT10 substrates. To test this hypothesis, the extent of NUB1-mediated degradation of the model FAT10 substrate HA-FAT10-DHFR [12] in the presence and absence of AIPL1 was examined. Post-transfection SK-N-SH cells were treated with the translation inhibitor cycloheximide (CHX) in order to assess FAT10-DHFR degradation. AIPL1 alone had no effect on FAT10-DHFR degradation, whereas NUB1 was observed to increase its degradation as expected (Figure 4A). Importantly, AIPL1 prevented FAT10-DHFR degradation in the presence of NUB1 (Figure 4A), and this effect was more pronounced upon treatment with proteasome inhibitor MG132, demonstrating its ability to antagonise the NUB1-mediated proteasomal degradation of a model FAT10 substrate. CHX-chase experiments were then performed to confirm the AIPL1-mediated effects on FAT10-DHFR degradation. Although NUB1 had stimulated the degradation of most detectable FAT10-DHFR by 2 hours CHX treatment, in the presence of AIPL1, FAT10-DHFR was still detectable after 2 hours (∼60% (64%±18) FAT10-DHFR levels remaining) and 4 hours (∼20% (24%±7) FAT10-DHFR levels remaining) of CHX treatment, demonstrating that AIPL1 significantly delayed the degradation of FAT10-DHFR in the presence of NUB1 (*p<0.050*, Wilcoxon signed-rank test; 2 h CHX treatment) (Figure 4B). In order to investigate whether the ability of AIPL1 to delay the NUB1-mediated degradation of FAT10-DHFR was relevant to LCA pathogenesis, the ability of the AIPL1 mutants A197P, C239R and G262S to hinder degradation of FAT10-DHFR was assessed. The AIPL1 mutants A197P and C239R, but not G262S, were defective in the ability to antagonise the degradation of FAT10-DHFR by NUB1 (Figure 4C). Because AIPL1 binds to both FAT10 and NUB1 to form a ternary complex, the ability of AIPL1 to act against the NUB1-mediated degradation of FAT10-DHFR could be related to its binding to NUB1 or FAT10-DHFR. To address this issue, NUB1 was expressed with wild type and mutant AIPL1 and their interaction was investigated by co-precipitation. Wild type and the AIPL1 G262S mutant efficiently co-precipitated NUB1. By contrast, the AIPL1 mutants A197P and C239R displayed severely reduced interactions with NUB1 (Figure 4D). Moreover, wild type AIPL1 and the mutants A197P, C239R and G262S were still competent in binding to FAT10-DHFR (Figure 4E). This suggests that the ability of AIPL1 to block the degradation of FAT10 substrates is dependent on the interaction of AIPL1 with NUB1, but not the FAT10 substrate.

### AIPL1 interacts with the FAT10 E1 activating enzyme UBA6

**Figure 5:**
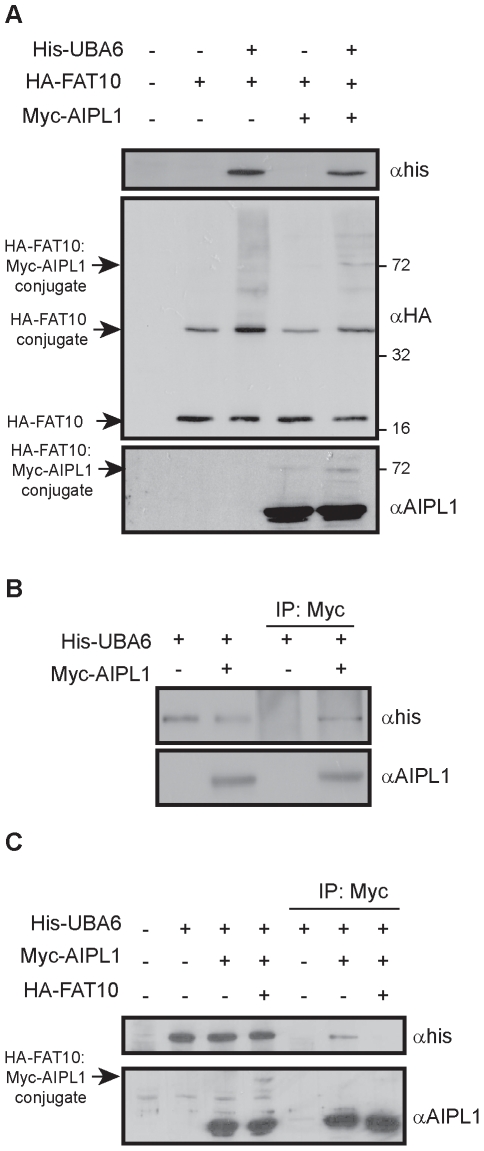
The FAT10 E1 activating enzyme interacts with AIPL1. Cells were transfected as indicated and subject to immunoprecipitation and immunoblot analysis. (A) Expression of the FAT10 E1 activating enzyme UBA6 increases the amount of FAT10-conjugated proteins including the covalent AIPL1-FAT10 conjugate. AIPL1 altered the profile of UBA6-dependent FAT10 conjugation. (B) UBA6 co-precipitated with AIPL1. (C) Co-precipitation of UBA6 with AIPL1 is abrogated in the presence of FAT10. The position of molecular weight markers is indicated in kilodalton (kDa).

The finding that AIPL1 binds to FAT10 and FAT10-modified proteins in addition to associating with NUB1 to antagonise degradation of a FAT10 model substrate led us to address whether AIPL1 may also interact with other components of the FAT10 conjugation system, specifically the E1 activating enzyme UBA6. His_6_-tagged UBA6 was co-expressed with FAT10 and led to a dramatic increase in high molecular weight FAT10-conjugated proteins as detected by western blot analysis (Figure 5A), consistent with its role as the FAT10 E1 activating enzyme. In addition, the levels of the AIPL1-FAT10 covalent conjugate were enhanced by UBA6 expression (Figure 5A). Interestingly, expression of AIPL1 altered the profile of high molecular weight FAT10 conjugates caused by UBA6 expression (Figure 5A), whereby several discrete FAT10-conjugate bands, including increased FAT10-modified AIPL1 (∼72 kDa) and additional bands of ∼82, 90 and 100 kDa, predominated over the characteristic FAT10 smear. Co-precipitation experiments revealed that UBA6 and AIPL1 were present in the same complex (Figure 5B), and because AIPL1 also interacts with FAT10 (Figure 2), we tested the effect of FAT10 expression on the interaction between AIPL1 and UBA6. Interestingly, FAT10 expression reduced the interaction between UBA6 and AIPL1 (Figure 5C), suggesting FAT10 and UBA6 may compete for a similar binding site on AIPL1.

## Discussion

The proteasome regulator NUB1 was identified as an interacting partner of the inherited blindness protein AIPL1 in a yeast two-hybrid screen [6], suggesting AIPL1 may influence retinal protein degradation pathways through modulating NUB1 function. The data presented here demonstrate that AIPL1 interacts with FAT10/FAT10-modified proteins, and acts to delay the NUB1-mediated degradation of a model FAT10 substrate. In addition, AIPL1 was shown to interact with the FAT10 E1 activating enzyme UBA6, which alongside its interaction with NUB1 and FAT10 suggest involvement of AIPL1 in the regulation of FAT10 function in the retina.

NUB1 was originally discovered due to its ability to interact with and promote the proteasomal degradation of NEDD8 and NEDD8-modified proteins [8], and subsequently was found also to have the same effect on FAT10 and FAT10-modified proteins [7]. As NUB1 was shown to accelerate the degradation of FAT10 four-fold more efficiently than NEDD8 [7], we tested if AIPL1 could affect the NUB1-mediated proteasomal degradation of FAT10ylated proteins. AIPL1 increased the steady-state levels of both monomeric FAT10 and FAT10-conjugated proteins in the presence of NUB1, suggesting it acts against NUB1-mediated proteasomal degradation of FAT10 substrates. Indeed, NUB1-mediated degradation of the model FAT10 substrate FAT10-DHFR was delayed in the presence of AIPL1. Furthermore, the pathogenic AIPL1 mutants A197P and C239R were both impaired in their ability to antagonise NUB1-mediated degradation, suggesting this function of AIPL1 may be relevant to LCA pathogenesis. While A197P and C239R cause LCA, the disease-causing status of G262S is less certain and this mutant was previously found not to be defective in a functional assay for NUB1 interaction and subcellular targeting [9], [27]. Consistent with these earlier findings, the AIPL1 mutants A197P and C239R, but not G262S, were defective in the ability to antagonise the degradation of FAT10-DHFR by NUB1. Interestingly, while the A197P and C239R mutants could still interact with FAT10-DHFR, they no longer interacted with NUB1, suggesting the antagonism of NUB1 function requires the direct binding of AIPL1 to NUB1. NUB1 is believed to be a facilitator of proteasomal degradation, whereby interactions between NUB1's UBL domain and the proteasome are essential for FAT10 degradation, possibly through inducing a conformational change in the 19S proteasome [15]. Therefore, the effects of AIPL1 on NUB1-mediated degradation may occur through AIPL1 binding to NUB1 and preventing NUB1 from productively associating with the proteasome to facilitate FAT10 degradation. Mutations that prevent association of AIPL1 with NUB1 and lose this ability may, therefore, contribute to disease by failing to prevent or delay the degradation of important, as yet undiscovered specific retinal FAT10 substrates.

Our data suggest that AIPL1, NUB1 and FAT10 form a ternary complex. In agreement with previously published work [27], we found that the C239R mutant of AIPL1 cannot bind NUB1. However, the presence of FAT10, which interacts with both AIPL1 C239R and NUB1, restores the ability of AIPL1 to co-precipitate NUB1, confirming the formation of a ternary complex. The binding site of AIPL1 on NUB1 has been reported to be C-terminal to the third UBA domain [27], consistent with the ability of NUB1 to simultaneously bind FAT10 and AIPL1. It is possible that FAT10 binds to NUB1 and AIPL1 through its distinct UBL domains to complete the ternary complex, although the precise binding interfaces between FAT10 and both NUB1 and AIPL1 remain to be determined. As the AIPL1 C239R mutant was able to interact with FAT10-DHFR, but not to antagonise its NUB1-mediated degradation, it is unclear what role the direct interaction between AIPL1 and FAT10 substrate plays in the ternary complex. One possibility is that AIPL1 may bind newly FAT10ylated substrates prior to their association with NUB1, thus preventing inappropriate NUB1-mediated degradation of the FAT10 substrate. It may be possible that a ternary complex would then exist until an appropriate signal initiates dissociation of AIPL1 to permit NUB1-mediated degradation of the FAT10 substrate, or release of the modified protein and removal of FAT10 by as-yet unidentified FAT10-specific proteases. Expanding the latter hypothesis is the possibility that FAT10 plays additional roles to protein degradation similar to other UBLs, whereby it may act as a signalling or subcellular localisation molecule. In this case, protection of the FAT10-modified protein from NUB1-mediated degradation by AIPL1 would be important for correct sorting or functional modulation of the modified protein. In any case, the future identification of retinal FAT10 substrates will allow a greater investigation into the role of AIPL1 and NUB1 in determining their fate.

AIPL1 has previously been shown to be essential for the biogenesis of rod PDE6 [2], [3], [4], and may cooperate with members of the Hsp70 and Hsp90 chaperone machineries to perform this function [5]. In the absence of AIPL1, PDE6 subunits are rapidly degraded by the proteasome [3], so it is plausible that certain PDE6 subunits may be direct substrates of FAT10ylation whose NUB1-mediated degradation is normally hindered by AIPL1. This scenario may fit with AIPL1 being involved in PDE6 quality control, whereby NUB1-mediated degradation of fully folded mature PDE6 is blocked by AIPL1, followed by removal of the FAT10 signal by unidentified FAT10-specific proteases, while any misfolded subunits would fail the quality control and be degraded. However, there is currently no evidence that PDE6 can be modified with FAT10, which could possibly reflect transient modification and rapid degradation or removal of FAT10. Alternatively, the role of AIPL1 in the FAT10 degradation pathway may represent a separate function of AIPL1, unrelated to its role as a specialised PDE6 chaperone.

In addition to delaying the degradation of a model FAT10 substrate, AIPL1 also co-precipitated the FAT10 E1 activating enzyme UBA6. Coupled to the fact that UBA6 enhanced the AIPL1-FAT10 covalent conjugate, this suggested AIPL1 may act as a non-canonical E2 conjugating enzyme in photoreceptors, whereby the FAT10-modification of AIPL1 may be via a thioester linkage. However, individually mutating all seven cysteine residues in AIPL1 did not abrogate the formation of the AIPL1-FAT10 conjugate and recombinant purified GST-AIPL1 was unable to form a thioester with FAT10 in the presence of recombinant E1 activating enzyme GST-UBA6 *in vitro* (data not shown), arguing against this possibility. Further work is therefore needed to establish exactly the nature of AIPL1's interaction with UBA6. It is possible that AIPL1 co-operates closely with the FAT10ylation machinery before it non-covalently binds to the new FAT10-modified substrate, protecting the substrate from NUB1-mediated degradation until appropriate spatial and temporal signals are received, for example, in the developing retina. Indeed, FAT10 itself is known to be cell cycle regulated [28], suggesting tight regulation of degradation of FAT10 substrates throughout the cell cycle is important. However, no E3 ligases or physiological substrates other than USE1 have thus far been reported in the literature to allow a more detailed investigation of the role of AIPL1 in this regard.

AIPL1 is only the fourth reported FAT10-interacting protein, after the spindle checkpoint protein MAD2 [17], NUB1 [7] and the histone deacetylase HDAC6 [21], and this is the first report to demonstrate that the FAT10 pathway is associated with inherited blindness. Interestingly, the association of FAT10 with HDAC6 occurred only upon proteasome inhibition, and caused microtubule-dependent recruitment of FAT10 to aggresomes, thus implicating FAT10 in lysosomal degradation pathways in addition to NUB1-mediated proteasomal degradation [21]. By contrast, the interaction of FAT10 with MAD2, which only occurred during mitosis, further suggested FAT10 involvement in the cell cycle [22]. In addition, FAT10 overexpression prevented the association of MAD2 with kinetochores and increased aneuploidy in HCT116 cells [22], evidence of a non-degradative role of FAT10. It would be interesting to test if the association of FAT10 and MAD2 could be regulated by AIPL1, which would have implications for cell cycle regulation in the developing photoreceptor.

The finding that AIPL1 appears to have a broad role in regulating the FAT10 pathway expands the role of AIPL1 as a specialised PDE6 chaperone to also having involvement in photoreceptor protein degradation pathways. The fact that mutations of AIPL1 were defective in antagonising NUB1-mediated degradation of a model FAT10 substrate imply this function of AIPL1 may be important in the pathogenesis of LCA. Further studies are required to find physiological retinal substrates for FAT10 modification, which will allow a more extensive characterisation of the role of AIPL1 in regulating the FAT10 pathway. In addition, the identity of these physiological substrates and how they are handled by AIPL1 prior to or as an alternative to degradation will lead to new insights in photoreceptor function, as well as expand our knowledge of the role of the FAT10 pathway in human biology.
